# Clinical and instrumental evaluation of primary and secondary glaucoma: Patterns and risk profiles

**DOI:** 10.6026/973206300222320

**Published:** 2026-04-30

**Authors:** Shivam Pandey, Kritika Upadhyay, Sanskriti Ukey, Shreyashi Tripathi, Shubhi Jain, Sachin Parmar

**Affiliations:** 1 Department of Ophthalmology, NSCB Medical College, Jabalpur, Madhya Pradesh, India; 2 Department of Ophthalmology, NandKumar Singh Chauhan Medical College Khandwa, Madhya Pradesh, India; 3 Department of Community Medicine V.K.S. Government Medical College, Neemuch, Madhya Pradesh, India

**Keywords:** Glaucoma, primary open-angle glaucoma, primary angle-closure glaucoma, secondary glaucoma, risk factors

## Abstract

Glaucoma is one of the leading causes of irreversible blindness that should be diagnosed and treated at an early stage. Hence, this cross-sectional study in the hospital setting was an evaluation of 270 patients with POAG, PACG, and secondary glaucomas with an evaluation of demographic, clinical, and instrumental parameters. The most prevalent subtype was POAG which was found in males aged between 51-60 years of age. The mean intraocular pressure was highest in PACG and the most common visual field defect was paracentral scotoma. Thus, we report the need to detect glaucoma early and treat it individually according to subtype and severity.

## Background:

Glaucoma is a chronic optic neuropathy with characteristic optic disc damage and corresponding visual field loss. It is classified as primary or secondary depending on whether a specific underlying cause is identified [1]. POAG is the most common subtype of POAG and PACG. Secondary glaucoma can result from trauma, inflammation, medications (including corticosteroids), or systemic disease that affects intraocular pressure [2]. The clinical evaluation of glaucoma includes history, slit-lamp biomicroscopy, gonioscopy, intraocular pressure measurement, optic nerve head assessment, and visual field testing [3]. Gonioscopy is best for anterior chamber angle assessment and angle-closure glaucoma diagnosis. OCT and HRT objective retinal nerve fibre layer loss and optic disc cupping support clinical decisions. Automaticperimetry detects glaucoma and functional visual field loss before symptoms appear [2]. Glaucoma candidates and acceleraters are identified by baseline risk profiling. POAG risk factors include older age, higher IOP, family history, African/Latino ethnicity, thinner central corneal thickness, longer axial length, and systemic factors like higher waist-to-hip ratio. Elevated IOP is the main modifiable factor linked to optic nerve damage, but prior ocular surgery, trauma, or inflammation can increase secondary glaucoma risk [1]. Some patients develop normal-tension glaucoma, where optic nerve damage occurs despite statistically normal IOP, suggesting a multifactorial pathogenesis. POAG is usually asymptomatic until late stages, with gradual peripheral field loss that may progress to tunnel vision [4]. Angle-closure glaucoma more often presents acutely with pain, redness, nausea, and rapid vision loss requiring urgent care, while many early glaucoma cases have few or no symptoms at diagnosis, contributing to underdiagnosis in population studies [5]. Glaucoma is the main cause of irreversible blindness in the world (15% of the global blindness), and the highest regional burden is felt in India (23.5% of the global glaucoma blindness). Clinical and instrumental assessment shows different patterns between primary and secondary glaucoma and each type has required differentiated diagnosis of clinical using tonometry, gonioscopy, visual field and structural imaging. Therefore, it is of interest to evaluate primary and secondary glaucoma and profiles risk factors and clinical features.

## Methodology:

This study was designed as a hospital-based, observational cross-sectional study conducted over a period of 12 months at a tertiary eye care center. The study protocol adhered to the tenets of the Declaration of Helsinki and received approval from the Institutional Ethics Committee. Informed consent was obtained from all participants prior to enrollment.

## Study population:

Patients diagnosed with primary and secondary glaucoma attending the glaucoma clinic were recruited consecutively. Inclusion criteria encompassed adults aged 18 years and above presenting with either primary open-angle glaucoma (POAG), primary angle-closure glaucoma (PACG), or secondary glaucoma of any etiology confirmed by clinical and instrumental examination. Exclusion criteria included patients with ocular media opacities precluding adequate examination, prior ocular surgery unrelated to glaucoma, and those unwilling to participate.

## Clinical evaluation:

A detailed ophthalmic history was recorded, including demographic data, family history of glaucoma, systemic diseases, and previous ocular interventions. Visual acuity was measured using a standardized Snellen chart. Slit-lamp biomicroscopy was performed to assess the anterior segment and rule out contributory pathologies. Intraocular pressure (IOP) was measured using Goldmann applanation tonometry under standard conditions. Gonioscopy was performed with a Goldmann 3-mirror lens to evaluate the anterior chamber angle status and classify glaucoma into open or angle-closure types. Fundus examination with a 90D lens was conducted to assess the optic nerve head for features consistent with glaucomatous damage, including cup-to-disc ratio, neuroretinal rim thinning and disc hemorrhages.

## Instrumental:

## Assessments:

Optical coherence tomography (OCT) was utilized to measure the retinal nerve fiber layer (RNFL) thickness and macular ganglion cell complex. Automated standard achromatic perimetry (SAP) was performed using a Humphrey Field Analyzer 24-2 program to evaluate the visual field defects.

## Data collection and analysis:

Baseline demographic, clinical, and instrumental data were recorded systematically. Risk factors such as age, IOP, family history, central corneal thickness (CCT), and axial length were documented. Patients were categorized into primary or secondary glaucoma groups for comparative analysis. Statistical analysis was conducted using SPSS software version 25. Descriptive statistics summarized demographic and clinical characteristics. Continuous variables were expressed as mean ± standard deviation, and categorical variables as frequencies and percentages. Comparative analyses between groups employed the chi-square test for categorical variables and t-test or Mann-Whitney U test for continuous variables based on data distribution. A p-value <0.05 was considered statistically significant. This comprehensive clinical and instrumental evaluation aimed to delineate patterns, characteristics, and baseline risk profiles in primary and secondary glaucoma to facilitate early diagnosis and targeted management strategies.

## Results:

Among 270 glaucoma patients, 160 (59.3%) were male and 110 (40.7%) were female (Table 1 - see PDF). The largest age group was 51-60 years (112, 41.4%), followed by 41-50 years (84, 31.1%), 61-70 years (65, 24.1%), and >71 years (9, 3.3%) (Table 2 - see PDF). POAG was the most common diagnosis (124, 45.9%), followed by PACG (72, 26.7%), secondary OAG (47, 17.4%), and secondary ACG (27, 10.0%); mean age was highest in POAG (57.57 ± 7.47 years) and PACG (56.39 ± 7.95 years), while secondary groups were younger (secondary OAG 51.62 ± 8.96 years; secondary ACG 51.41 ± 6.10 years), with mean IOP ranging from 23.94 ± 3.84 mmHg (POAG) to 26.41 ± 1.31 mmHg (secondary ACG), and mean CCT ranging from 556.70 ± 19.89 µm (secondary ACG) to 569.96 ± 11.37 µm (secondary OAG) (Table 3 - see PDF). Visual acuity distributions varied across groups, with POAG and PACG showing a wider spread across acuity categories, secondary OAG clustering mainly between 6/9 and 6/18, and secondary ACG showing more patients in poorer acuity categories such as 6/18-6/36 (Table 4 - see PDF). On visual field testing, 74 (27.4%) had normal fields, while 196 (72.6%) had defects-most commonly paracentralscotoma (70, 25.9%), followed by Seidel's scotoma (46, 17.0%), arcuatescotoma (41, 15.2%), double arcuatescotoma (24, 8.9%), and tunnel vision (15, 5.6%) (Table 5 - see PDF).

## Discussion:

This study sheds light on glaucoma patients' demographic, clinical, and instrumental traits. The prevalence was higher in males (59.3%) than females (40.7%) among 270 patients. This male predominance is consistent with Parab *et al.* [6], who found a higher proportion of male glaucoma patients, although gender distribution may vary with geographic and ethnic factors, as Nakaniida *et al.* [7] found female predominance. Genetic, hormonal, and healthcare- seeking differences may explain why men have more glaucoma. This study found the highest prevalence (41.4%) in patients aged 51-60, supporting the idea that glaucoma prevalence rises with age. Lee *et al.* found that age is a risk factor for glaucoma development and progression [8]. The lower representation of patients over 70 may reflect decreased life expectancy or under diagnosis. POAG was the most common subtype (45.9%), followed by PACG (26.7%) and secondary glaucomas. This distribution supports Kumar *et al.* and others' findings that POAG accounts for most glaucoma cases, along with PACG and secondary glaucomas [9]. PACG patients had a higher mean intraocular pressure (IOP) (26.40 mmHg) than POAG patients (23.94 mmHg), consistent with angle-closure glaucoma's pathophysiology of acute or chronic angle obstruction. CCT differences between groups confirm that PACG has thicker corneas than POAG. POAG patients had a wider range of VA, with many maintaining moderate vision (6/18), supporting the idea that POAG is insidious and slow-progressing. Secondary Angle-Closure Glaucoma patients had worse VA, indicating more severe or rapid glaucomatous damage, which is consistent with secondary glaucoma often associated with underlying ocular pathologies. Takahashi *et al.* found that ageing, corneal biomechanics, and ocular blood flow affect glaucoma patients' visual acuity [10]. Paracentralscotoma was the most common visual field defect in 25.9%, followed by Seidel's and arcuatescotomas. This matches POAG's early functional impairment, which involves central vision before peripheral constriction. Advance defects like tunnel vision in 5.6% of glaucoma patients demonstrate its natural progression and emphasise early detection and treatment. Other epidemiological and clinical studies show that demographics and baseline clinical parameters affect glaucoma subtype presentation and progression. Family history and ethnicity are non-modifiable susceptibility factors, as noted in previous reviews. IOP, CCT and detailed visual field assessment are essential for glaucoma stratification and management, according to the study.

## Conclusion:

The understanding of demographic and clinical profiles of glaucoma patients, highlighting the diversity within glaucoma subtypes regarding risk factors, visual function, and disease severity is highlighted. It emphasizes the necessity of comprehensive clinical and instrumental evaluations for timely diagnosis and tailored treatment strategies. Future longitudinal investigations should focus on the impact of early therapeutic interventions on the progression of glaucoma across diverse populations.
